# Impact of pulse sequence, analysis method, and signal to noise ratio on the accuracy of intervertebral disc *T*_2_ measurement

**DOI:** 10.1002/jsp2.1102

**Published:** 2020-06-26

**Authors:** Kyle D. Meadows, Curtis L. Johnson, John M. Peloquin, Richard G. Spencer, Edward J. Vresilovic, Dawn M. Elliott

**Affiliations:** ^1^ Biomedical Engineering University of Delaware Newark Delaware USA; ^2^ National Institute on Aging, National Institutes of Health Baltimore Maryland USA; ^3^ UPMC Orthopaedic Surgery Lancaster Pennsylvania USA

**Keywords:** degenerative disc disease, intervertebral disc, MRI, Rician noise, *T*_2_

## Abstract

Noninvasive assessments of intervertebral disc health and degeneration are critical for addressing disc degeneration and low back pain. Magnetic resonance imaging (MRI) is exceptionally sensitive to tissue with high water content, and measurement of the MR transverse relaxation time, *T*_2_, has been applied as a quantitative, continuous, and objective measure of disc degeneration that is linked to the water and matrix composition of the disc. However, *T*_2_ measurement is susceptible to inaccuracies due to Rician noise, *T*_1_ contamination, and stimulated echo effects. These error generators can all be controlled for with proper data collection and fitting methods. The objective of this study was to identify sequence parameters to appropriately acquire MR data and to establish curve fitting methods to accurately calculate disc *T*_2_ in the presence of noise by correcting for Rician noise. To do so, we compared *T*_2_ calculated from the typical monoexponential (MONO) fits and noise corrected exponential (NCEXP) fits. We examined how the selected sequence parameters altered the calculated *T*_2_ in silico and in vivo. Typical MONO fits were frequently poor due to Rician noise, and NCEXP fits were more likely to provide accurate *T*_2_ calculations. NCEXP is particularly less biased and less uncertain at low SNR. This study showed that the NCEXP using sequences with data from 20 echoes out to echo times of ~300 ms is the best method for calculating *T*_2_ of discs. By acquiring signal data out to longer echo times and accounting for Rician noise, the curve fitting is more robust in calculating *T*_2_ despite the noise in the data. This is particularly important when considering degenerate discs or AF tissue because the SNR of these regions is lower.

## Graphical abstract

*T*_2_ measurement is susceptible to inaccuracies due to Rician noise, *T*_1_ contamination, and stimulated echo effects. These error generators can all be controlled for with proper data collection and fitting methods. The objective of this study was to identify sequence parameters to appropriately acquire MR data and curve fitting methods to accurately calculate disc *T*_2_.

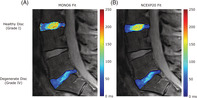

## 1 INTRODUCTION

Noninvasive assessment of intervertebral disc degeneration is critical for addressing low back pain, for evaluating treatment efficacy in patients, and for evaluating preclinical animal models of disc disorders. Magnetic resonance imaging (MRI) is exceptionally sensitive to tissue with high water content. For this reason, MRI is widely used for the disc with grading schemes based on structure and signal intensity from *T*_2_‐weighted (*T*_2_w) images. The contrast provided by *T*_2_w MRI is particularly well‐suited for structural evaluation because it provides contrast between a bright nucleus pulposus (NP), a dark annulus fibrosus (AF), and a dark vertebral body at the inferior and superior boundaries of the disc. In the Pfirrmann grading scheme and others, structural features are evaluated and graded based on NP brightness and uniformity, NP‐AF distinction, and disc height.^1^ Unfortunately, such evaluation is highly subjective and is nonquantitative in terms of degree of pathology. Further, the phenotypes of human disc degeneration are a continuum that are too complex to be categorized by the five Pfirrmann grades. For these reasons, measurement of the MR transverse relaxation time, *T*_2_, has been proposed and applied as a quantitative, continuous, and objective measure of disc degeneration that is linked to the water and matrix composition of the disc. *T*_2_ is defined as the time constant of the decay, or relaxation, of the transverse signal and is calculated from a *series* of *T*_2_w images.^2^, ^3^ *T*_2_ is longer in healthy discs, which have higher water content and water mobility, and decreases as the disc degrades and loses proteoglycan and water content.

**FIGURE 1:**
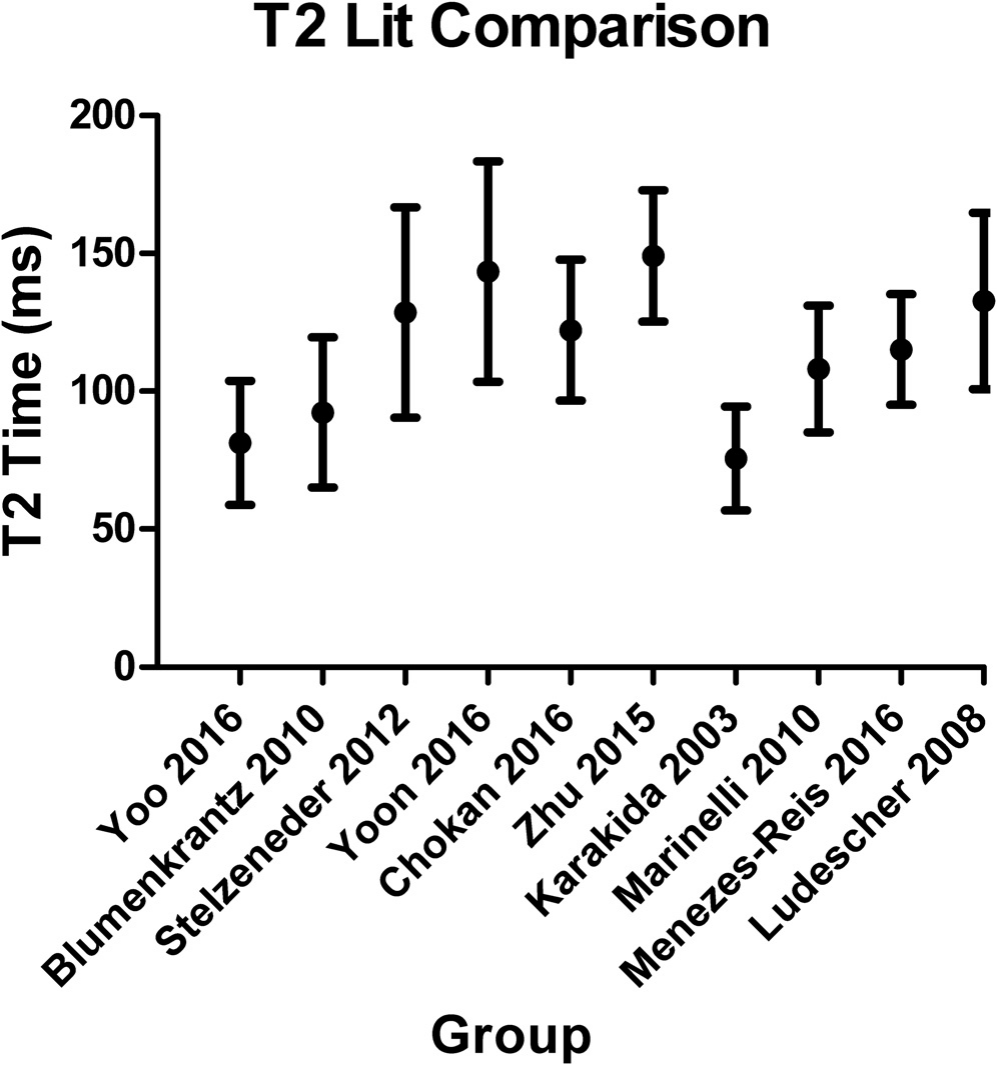
Reported *T*_2_ for healthy discs from volunteers without back pain. Despite sampling a similar population, reported mean T_2_ vary by up to a factor of two across studies, ranging from a mean of 76 to 149 ms. Dots and bars represent mean +/− SD

**FIGURE 2:**
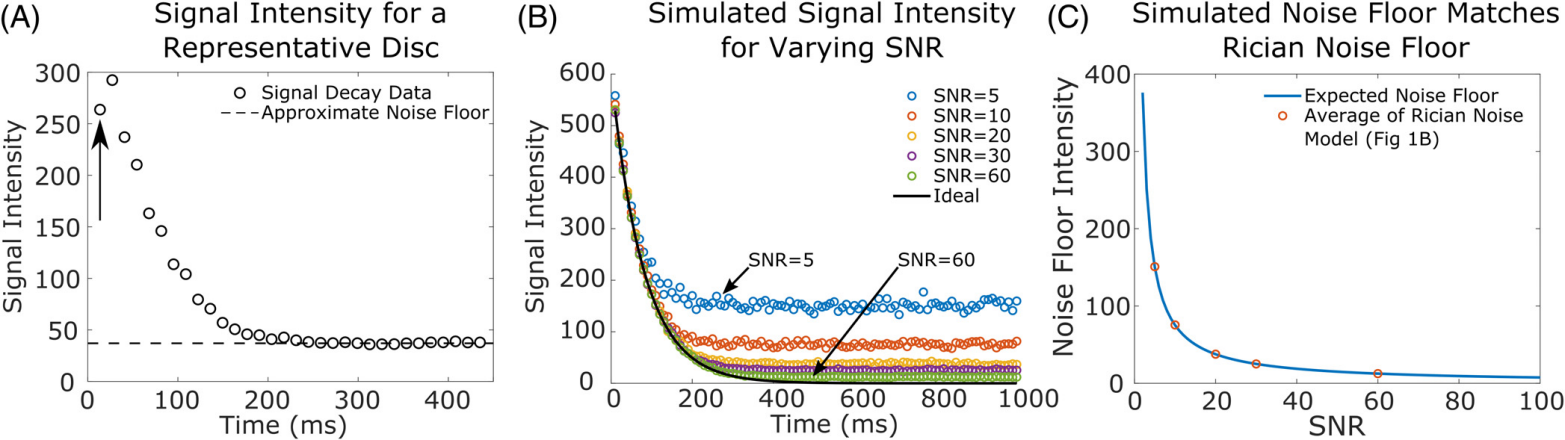
(A), Signal intensity as a function of echo time for a representative disc, where each data point represents a signal intensity measured at a *TE*. It can be observed that the signal decays to a nonzero noise floor (dashed line). Note that the first echo (arrow) has lower signal because of the stimulated echo effect affecting all subsequent echoes. (B), The effect of the amount of SNR on the noise floor for simulated Rician noise at varying SNR levels. Without noise (solid line) the signal intensity decays to 0, but as the SNR decreases, the noise floor level increases. (C), The expected noise curve vs SNR. The noise floor for each SNR level in (B) is shown and matches the expected curve almost exactly

*T*_2_ measurement is becoming more widely used as a measure of disc degeneration,^4^, ^5^, ^6^, ^7^, ^8^, ^9^, ^10^, ^11^, ^12^, ^13^, ^14^, ^15^ however, there are limitations in its application that can make comparisons across studies problematic and, in the worst case, provide inaccurate *T*_2_ values. First, a wide variety of MR sequences and calculation methods have been implemented, leading to a large range of reported average *T*_2_ from 75 to 150 ms for similar populations of young, healthy, nondegenerative discs (Table 1, Figure 1). This is likely due in large part to the variability in the sequence parameters (*TR*, *TE*, and number of echoes) which are sometimes outside the recommended range based on disc material parameters (*T*_1_ and *T*_2_), as described in the next section. In addition, greater accuracy is often obtained by excluding the first echo in data fits,^16^ as seen in some cartilage studies.^17^ The first echo is excluded because it is the only data point for which the phenomenon of stimulated echoes does not occur, and therefore it follows a different decay than the subsequent data points^16^, ^18^ (Figure 2A). It is assumed that most published work has excluded this first echo, but it is not always explicitly stated and researchers new to the field may not be aware of this limitation, which is ultimately based on the unavoidable inhomogeneity of RF pulse amplitude throughout the sample. Finally, MR imaging is susceptible to noise that can corrupt fits of the *T*_2_ decay curve. Notably, Rician noise results in an altered signal decay curve that decays to a nonzero value called the “noise floor” (Figure 2A), which causes error in *T*_2_ calculation because the fit equation assumes a monoexponential signal decay to zero. The impact of this noise is that the calculated *T*_2_, which is a material parameter, can be inaccurate. This effect is influenced by the signal to noise ratio (SNR) and whether the number of echoes acquired is sufficient so that the noise floor is approached during signal decay. This effect can be addressed through careful design of data acquisition and modeling of noise characteristics as will be shown in this study.

**TABLE 1:** Sequence parameters used to acquire data for *T*_2_ calculations shown in Figure 1

Citation	*TR* (ms)	First *TE* (ms)	Last *TE* (ms)	# echoes	Average *T*_2_ (ms)
Yoo, 2016	1836	6	38.1	4	81.30
Blumenkrantz, 2010	Not reported	9.6	77.2	7	92.30
Stelzeneder, 2012	1200	13.8	82.8	6	128.60
Yoon, 2016	120	9.9	89.1	9	143.47
Chokan, 2016	2000	13	103	8	122.10
Zhu, 2015	2000	13.9	111	8	149.10
Karakida, 2003	2000	30	120	4	75.56
Marinelli, 2010	2000	9	144	16	108.00
Menezes‐Reis, 2016	3000	20	160	8	115.10
Ludescher, 2008	3000	9	288	32	132.80
Recommended from present study	≥3000		≥300		

*Note:* There is a large range of sequence parameters used, which lead to wide variations in reported *T*_2_.

A noise‐corrected exponential (NCEXP) has successfully accounted for Rician Noise in cartilage and has been used to calculate accurate *T*_2_ ^19^, ^20^; however, it has not been applied to the disc. It is critical to address the effect of the Rician noise in the disc because the disc signal decays to low SNR, m consideration of the Rician distribution of noise important. Moreover, the disc loses hydration and signal intensity with degeneration, which will decrease the initial SNR and exacerbate the role of Rician noise. Therefore, the objective of this study is to identify appropriate sequence parameters to acquire MR data and to establish curve fitting methods to accurately calculate disc *T*_2_ in the presence of noise by correcting for Rician noise. To do so, we compared *T*_2_ calculated from the typical monoexponential (MONO) fits and noise corrected exponential (NCEXP) fits and examined how the selected sequence parameters altered the calculated *T*_2_. We also evaluated how *T*_2_ is affected by performing fits of intensity data averaged over a region‐of‐interest (ROI) to suppress noise vs performing fits at each individual voxel. Based on these results, we recommend methods to select sequence parameters and to calculate *T*_2_ for the disc to address the effect of Rician noise and the low signal intensity at long echo times, particularly for the degenerating disc.

## 2 THEORY

Determining *T*_2_ requires a longer and more complex imaging sequence than acquisition of the single *T*_2_w image that is used for grading. However, the advantage of this sequence is that it gives quantitative material property information that is less susceptible than single *T*_2_w signal intensity to environmental factors, scanner strength, magnetic field inhomogeneities, and subject traits such as body weight. The signal intensity in each voxel in a conventional MR image is often modeled as

(1) Signal intensity∝H∙1−e−TRT1∙e−TET2

where the repetition time, *TR*, is the time between individual spin excitation pulses, and the echo time, *TE*, is the time of echo occurrence after the initial excitation pulse. *TR* and *TE* are user‐defined input parameters, while the *T*_1_ and *T*_2_ relaxation times are material properties, with *T*_1_ being the spin‐lattice, or longitudinal, relaxation time; *H* is the number of protons in a voxel. Although Equation (1) omits the dependence of signal intensity on a number of nonmodeled effects (e.g., pulse errors, diffusion, chemical exchange, nonmonoexponential relaxation behavior), it correctly describes the dominant dependences on *TR* and *TE* in the noise‐free case. Clinical MR images are formed from the absolute magnitude of the signal (i.e., signal intensity being the square root of the sum of the squares of the signal acquired in real and imaginary channels); this is a crucial concept for the understanding of Rician noise as described below. See References 21, 22 for an overview of MRI fundamentals and definitions.

The *TR* selected for the sequence is the dominant determinant of the total scan time. In *T*_2_w MRI, *TR* is often selected to minimize *T*_1_ weighting in the acquired signal (i.e., with *TR* satisfying e−TRT1→0), so that residual image intensity weighting is primarily dependent on *T*_2_, or more precisely, on the ratio *TE*/*T*_2_. Ideally, *TR* would be ≥ *T*_1_*5 in order to achieve <1% *T*_1_ weighting, however since disc *T*_1_ is approximately 1200 ms,^23^ this would lead to very lengthy scans. Thus, the selection of *TR* is a compromise between scan duration and the desired limit e−TRT1→0.

The echo time in Equation (1), *TE*, defines the delay between an initial excitation pulse and signal acquisition at the peak of the subsequent echo, with a refocusing pulse applied between these at a time *TE*/2 after spin excitation. To determine *T*_2_, signal intensity is measured across a series of echo times, which are conventionally multiples of a minimum *TE*. This means that samples are obtained at times n*TE following excitation, with n ranging from one to some large value defining the number of echoes acquired. From Equation (1), it is clear that signal amplitude will decrease as an exponential function of *TE*, with time constant *T*_2_. Note that this assumes a monoexponential signal equation, which is commonly used when curve fitting signal decay data to determine *T*_2_ in disc. The minimum *TE* used should be small enough to permit signal acquisition from rapidly relaxing tissue, and the number of echoes should be large enough to permit near complete signal decay during the echo train. We chose 85% signal decay as a criterion, as further decay would generally result in signal at the noise floor. Since disc *T*_2_ is approximately 150 ms in healthy NP, the maximum *TE* should be at least 300 ms, to capture 85% of the signal decay. Because *TR* is much longer than *TE*, multi‐echo MRI sequences can collect many TEs without any cost to overall imaging time.

Rician noise causes an alteration in the shape of the signal decay along with causing the signal to decay to a nonzero value. This Rician noise can be modeled to calculate more accurate *T*_2_ values. Rician noise occurs in the MR signal magnitude due to the Gaussian noise characteristics of the real and imaginary signals acquired to compute the magnitude image. The Rician probability distribution is given by

(2) $$\begin{array}{c} p_{M}\left(M\right)=\frac{M}{\sigma^{2}}e^{-\frac{M^{2}+S^{2}}{2\sigma^{2}}}I_{0}\left(\frac{S∙M}{\sigma^{2}}\right) \end{array}$$

where *M* is the measured signal intensity, *S* is the signal intensity without noise, *σ* is the SD of the Gaussian noise in the real and imaginary components, and *I*_0_ is the modified zeroth order Bessel function of the first kind.^24^ A key characteristic of the Rician distribution is that at high SNR it approximates a Gaussian distribution. However, when the SNR is low, the noise associated with the signal is no longer Gaussian because taking the magnitude of real and imaginary components is a nonlinear function. At the extreme of SNR = 0 (no tissue signal, only noise signal) the measured MR signal takes on a nonzero mean value (Rayleigh distribution) that we call the noise floor (dashed line in Figure 2A).^20^, ^24^ This phenomenon was previously studied with simulation and articular cartilage samples using a noise corrected exponential (NCEXP) that fit the signal while incorporating the expected value of the Rician noise, thereby more accurately determining *T*_2._ ^19^ The expected noise corrected signal intensity is given by

(3) $$\begin{array}{c} \text{Signal intensity}\left(S,\sigma \right)=\sqrt{\frac{\pi \sigma^{2}}{2}}e^{-\alpha }\left(\left(1+2\alpha \right)I_{0}\left(\alpha \right)+2\alpha I_{1}\left(\alpha \right)\right) \end{array}$$

where α=S2σ2, and *I*_1_ is the modified first order Bessel function of the first kind.

## 3 METHODS AND RESULTS

### 3.1 Simulation of Rician noise and dependence on SNR

We first confirmed that simulated Rician noise recapitulated the noise floor observed in the disc (Figure 2A) and that we could model the signal intensity of the noise floor as a function of the SNR.

Using MATLAB (MathWorks), an ideal monoexponential decay was generated with an initial signal of 600 (S_0_) and *T*_2_ of 80 ms.

(4) STE=S0*e−TET2

Rician noise was simulated by adding Gaussian noise to both the real and imaginary components of the ideal monoexponential decay. The resulting signal intensity, calculated as the magnitude of the real and imaginary components, was averaged for 100 simulated voxels to mimic the size of an ROI in an in vivo measurement. The σ of the Gaussian noise was given by S_0_/SNR, and this simulation was repeated for SNR ranging from 5 to 60. The resulting signal was plotted over time (every 10 ms out to 1000 ms, that is, *TE* = 10 ms with 100 echoes) and compared qualitatively to the observed signal intensity decay and noise floor from an in vivo disc (Figure 2A).

The noise floor of the simulated signal was calculated as the average signal of the last 50 points for each simulated SNR (Figure 2B). These values were then plotted as a function of SNR for the 5 SNR values used (Figure 2C, circles). The expected relationship between noise floor and SNR (mean of Rayleigh distribution) is also plotted (Figure 2C, curve), based on the following equation

(5) Expected noise floor=σ*π2=S0SNR*π2

where the initial signal, *S*_0_, was equal to 600.

Our simulation of Rician noise as a function of SNR confirms b the noise floor observed in the disc (Figure 2A,B) and the expected noise floor as derived from MR physics (Figure 2C). The signal intensity with simulated Rician noise (Gaussian noise applied to the real and imaginary components) demonstrated a nonzero noise floor that depends on the SNR (Figure 2B) and is consistent with in vivo disc imaging data (Figure 2A). The signal intensity of the noise floor is greater with a low SNR of 5 and is negligible with a high SNR of 60 (Figure 2B). Moreover, when the noise floors of the simulated signal intensity values are compared to the curve of the expected noise floor, a perfect overlap is observed that demonstrates the accuracy of our Rician noise simulation (Figure 2C).

### 3.2 Simulation of *T*_2_ sensitivity to fitting model and number of echoes

Signal intensity data with simulated noise was used to demonstrate the sensitivity of the calculated *T*_2_ to (a) the degree of noise (low vs high SNR), (b) the model used to calculate *T*_2_ (monoexponential or noise corrected exponential), and (c) the effect of the number of echoes in the dataset (simulating the number of *TE* chosen for the MR sequence during data acquisition). Data were simulated using physiological *T*_2_ for an unhealthy disc (*T*_2_ = 80 ms) and *TE* = 13.6 ms with 20 echoes out to 272 ms to reflect typical MR data acquisition. Low and high SNR data were both simulated (SNR = 5 and 30) using the definition SNR=S0σ. For each SNR, 100 voxels of signal intensity data were simulated, to represent an ROI, and these signal intensities were averaged and plotted against time to be used for curve fitting to calculate *T*_2_.

*T*_2_ was then calculated using two different curve fits: typical mono‐exponential (MONO) and noise corrected exponential (NCEXP).^19^ As described previously, MONO represents the decay to zero which is typically used in the field, and NCEXP represents the corrected signal model that includes Rician noise and the resulting nonzero noise floor. The MONO fit was calculated using MATLAB's built in *fit* function under default options using a monoexponential curve (Equation 4). Two parameters, S_0_ and *T*_2_, were allowed to vary and were unbounded in the fit. The parameters producing the lowest residual error between data and curve fit were used. The NCEXP fit was calculated using nonlinear least squares methods using MATLAB's *fmincon* function with a multi‐start approach using 100 randomized initial guesses. The NCEXP curve fit has three parameters: *T*_2_, S_0_, and *σ*. Their lower and upper bounds were set to: [0 300 ms], [100 1000], and [20 80], respectively. The fit with the lowest residual error was taken as the best fit, and its parameters reported.

The curve fit procedure was repeated multiple times with different numbers of echoes in the dataset to simulate differing number of TEs chosen during data acquisition. Curve fitting started with just the first four echoes (acquisition to 54.4 ms) and was repeated, increasing the number of echoes by one each time, up 20 echoes (acquisition to 272 ms). Note that the first echo (*TE* = 13.6 ms) was ignored in the fitting to mimic how real data should be handled. The calculated *T*_2_ and goodness of fit (mean squared error, MSE) were plotted vs the number of echoes used for both model types (MONO and NCEXP) and for both low and high SNR (5 and 30) to illustrate the impact of number of echoes, correction for noise, and SNR on the accuracy of calculated *T*_2_ and fit quality.

**FIGURE 3:**
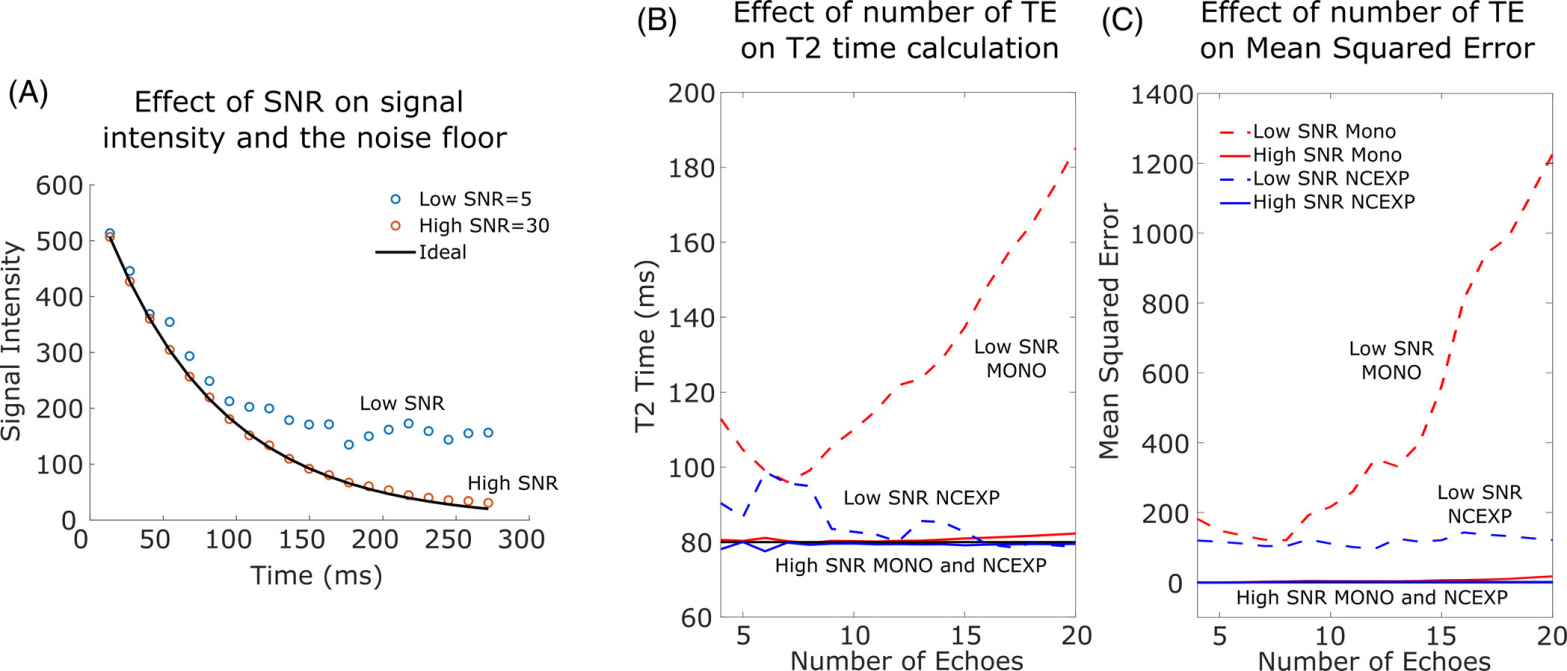
Simulation of the effect of SNR on the noise floor and the effect of the fit model on the calculated *T*_2_ for a high SNR of 30 and a low SNR of 5. A, The high SNR disc follows the ideal decay closely out to 272 ms, but the low SNR disc hits a nonzero noise floor. For each curve fitting method, monoexponential (MONO) or noise corrected exponential (NCEXP), the calculated, B, *T*_2_ and C, MSE are shown as a function of number of echoes in the fit. For a MONO fit, the high SNR disc maintains a near perfect 80 ms *T*_2_ and MSE below 20 across all echo train lengths, but the low SNR disc has inaccurate *T*_2_ values and worse fit at all number of echoes. For the NCEXP, *T*_2_ and MSE are closer to ideal than the monoexponential for the low SNR disc, but slightly inaccurate at low number of echoes. The high SNR disc is well fit with the NCEXP giving an accurate *T*_2_ across all number of echoes

The signal intensity data simulated with and without noise for a material with 80 ms *T*_2_ (Figure 3A) demonstrates that a low SNR alters signal decay and generates a nonzero noise floor, while a larger SNR of 30 is close to the ideal signal decay. Indeed, for a large SNR of 30, both curve fitting methods produce a calculated *T*_2_ near the expected value of 80 ms and a goodness of fit (MSE) near the ideal value of 0 (Figure 3B,C). Specifically, MONO *T*_2_ is within 3 ms for all echo train lengths (max MSE = 18), and NCEXP *T*_2_ is within 2 ms for all echo train lengths (max MSE = 3). Therefore, high SNR does not require noise correction or long echo trains; however, this high SNR is not typically achievable in practice. With a more realistic SNR of 5, the effect of the model and number of echoes is large. For the low SNR of 5, when fewer than 6 echoes are used in the fit, the MONO fit overestimates *T*_2_ due to the impact of noise on the low number of data points in the fit. Importantly, for low SNR, the MONO fit never matches the expected *T*_2_ of 80 ms regardless of the number of echoes used because of the altered decay curve and noise floor (Figure 3B, red dashed). The MONO fit is closest to the expected 80 ms at 7 echoes (approximately 95 ms), but thereafter increases with increasing number of echoes, reaching a maximum calculated *T*_2_ of 186 ms at 20 echoes or a >2× overestimation of the expected *T*_2_. Accordingly, MONO has the highest MSE at all echo train lengths and has a maximum of 1226 with 20 echoes in the echo train (Figure 3C, red dashed). In contrast, NCEXP is within 6 ms of the expected *T*_2_ for echo trains with 8 or more echoes (Figure 3B, blue dashed) and has a much lower MSE than MONO at longer echo train lengths, with a maximum MSE of 143 with 16 echoes in the echo train (Figure 3C, blue dashed). Overall, the MONO fit has large errors that depend on the number of echoes, while NCEXP matches expected values and the number of echoes has less impact.

### 3.3 Sensitivity of *T*_2_ to fitting model and number of echoes for in vivo human disc data

To evaluate the sensitivity of *T*_2_ to the fitting model and number of acquired echoes, in vivo spine imaging was performed, and disc signal intensity was fit with two models and two different number of echoes. Lumbar spines from healthy volunteers with no history of back pain were scanned under IRB approved protocols after providing informed written consent (n = 8, 24‐31 years old). All lumbar discs from each spine were imaged and included in the analysis for a total sample size of 40 discs (n = 35 Pfirrmann grade I‐II, n = 5 grade III‐IV). To minimize variation, each scan was acquired at 8 am (after a full night's sleep) with minimal activity prior to scanning and with the subject laying supine at the MRI facility for at least 45 minutes prior to scanning. A single sagittal slice CPMG sequence was used to collect *T*_2_ data on a 3 T Siemens Magnetom Prisma scanner.^3^ Sequence parameters included: FoV = 165 × 220 mm, *TR* = 3000 ms, *TE* = 13.6, 27.2, …, 272 ms (20 total echoes), voxel size 0.57 × 0.57 × 5.00 mm. *TR* was selected to minimize the contribution of *T*_1_ in the measured signal (see Section 2 ^23^) and 20 echoes were acquired to obtain several data points for evaluating model fits. Scan time was 14:29 minutes.

Image analysis was performed with an in‐house code by first creating a circular ROI in the middle of the NP and calculating the average signal intensity in the ROI for each echo, analogous to simulations above. The ROI included 80 to 120 voxels, depending on disc size. Curve fits were then performed on these average intensities to find *T*_2_. The first echo was excluded for curve fitting.^16^

Signal intensity data were fit using two different models (monoexponential and noise corrected exponential) and two different number of echoes (6 and 20), resulting in the following four groups for comparison: MONO6, MONO20, NCEXP20, and NCEXP6 defined based on fit method and number of echoes collected. The cases with 6 collected echoes are similar to the shorter sequences used in several published studies reporting disc *T*_2_, and the case with 20 collected echoes represents a larger number of data points in the fit that captures nearly the entire decay and likely extends into the noise floor. It is important to note that only one MRI sequence was acquired per subject to obtain the data. The same data is being analyzed for each fit case, with a different curve fit and number of echoes being considered. Unlike in simulation, where SNR could be specified, the SNR of in vivo scans cannot be easily controlled but is the result of several factors including the subject, the disc characteristics, the MR system, and the selected sequence.

Results were tested for normality using a Jarque‐Bera test, and failed to reject the null hypothesis that the data was normally distributed.^25^ To test for the effect of curve fit on the calculated *T*_2_, a 2‐way ANOVA was run followed by post‐hoc matched pair t‐tests between all pairings with a significance level set at alpha = 0.05.

**FIGURE 4:**
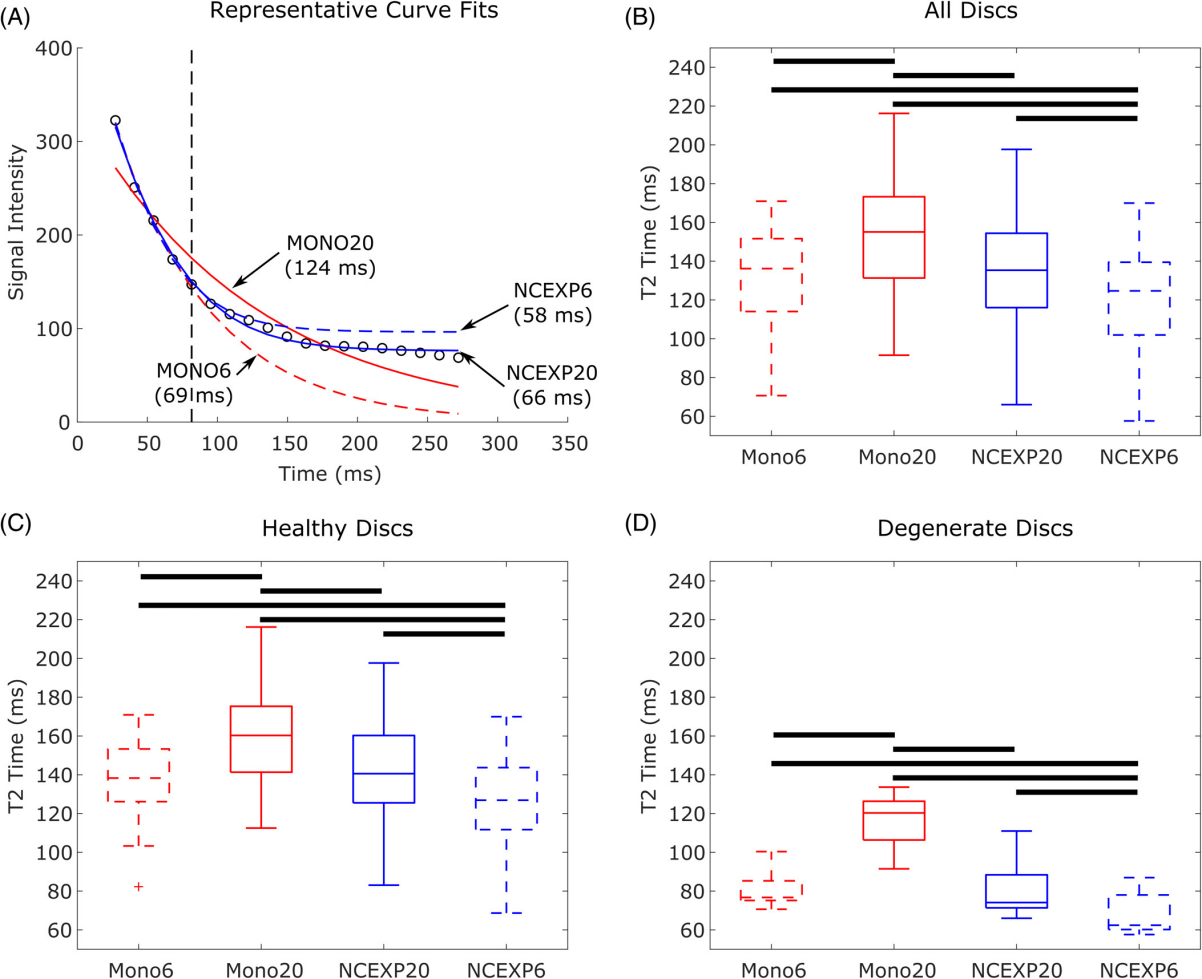
A, Representative dataset for a degenerated disc showing all four curve fitting methods. Vertical dashed line represents the last echo used for fitting for MONO6 and NCEXP6 fits (first echo is ignored and not shown) and the rest of the curve is extrapolated. NCEXP fits look better than MONO fits. NCEXP20 and MONO6 give very similar *T*_2_, but MONO6 is a bad fit. B‐D, calculated *T*_2_ for, B, all discs (n = 40), C, healthy discs (n = 35), and D, degenerate discs (n = 5). MONO20 is higher than other measures and likely overestimated because of the noise floor. NCEXP6 is lower than others and likely underestimated because of insufficient data points to predict the noise floor. For all cases MONO6 and NCEXP20 are not different from each other even though the fits for MONO6 are not as strong as those for NCEXP20. Boxes represent median, 25th, and 75th percentiles. Whiskers extend to most extreme data point that is not considered an outlier (+ symbol)

Representative data for a Pfirrmann grade IV disc is plotted vs time with fits for MONO and NCEXP curves with 6 or 20 echoes and their respective *T*_2_ (Figure 4A). The dashed vertical line represents the last echo in the fit for the MONO6 and NCEXP6 curve fits (the first echo is not shown). As with the simulated data, the MONO20 curve fit overestimates *T*_2_ (124 ms) and the fit is not very strong (MSE = 521). NCEXP20 and MONO6 give very similar *T*_2_ (66 and 69 ms, respectively). The goodness of fit for MONO6 is great when considering the first six echoes (MSE = 27), but when compared to the full data set the fit is much worse (MSE = 1679) than the fit for NCEXP20 (MSE = 20) meaning it does not accurately describe the full data. This is to be expected as the MONO6 fit only uses the first six echoes and assumes that the signal will decay monoexponentially to zero. The NCEXP6 *T*_2_ is slightly lower than NCEXP20 and MONO6 (58 ms), and while the NCEXP6 fit is good for 6 echoes (MSE = 17), it does not fit the whole data set (MSE = 208) as well as NCEXP20.

When comparing the 4 different calculation methods across all 40 discs (Figure 4B), the same pattern emerges as with the representative disc (Figure 4A). Two‐way ANOVA showed significant differences by both factors: echo and curve fit (*p* < .001). MONO6 and NCEXP20 give similar *T*_2_, 131.12 ± 27.86 ms and 133.66 ± 31.25 ms, respectively, and are not different from one another, despite NCEXP20 fitting better than MONO6 (*p* > .05). MONO20 gives *T*_2_ that are significantly higher than all other measures (154.4 ± 29.28 ms, *p* < .001) and likely overestimates *T*_2_ due to sampling of the noise floor. NCEXP6 gives values that are significantly lower than all other measures (119.01 ± 28.85 ms, *p* < .001) and likely underestimates *T*_2_ due to incorporation of an insufficient number of data points (Figure 4B). This finding is the same when the discs are separated into nondegenerate (n = 35, Figure 4C) and degenerate (n = 5, Figure 4D) discs. The error introduced by using the MONO20 fit in healthy discs is approximately 20 ms compared to NCEXP20, while in the degenerate discs the difference is much larger at approximately 40 ms. It should be noted that NCEXP20 predicted an average SNR of 10.07 (min 4.65, max 29.26) across all 40 discs, as calculated from the fitting parameters using SNR=S0σ.

### 3.4 Accuracy of MONO6 and NCEXP20

To determine which fitting method was most accurate, simulations were performed to find the bias and uncertainty from MONO6 and NCEXP20 curve fitting methods. The MONO20 and NCEXP6 fitting methods were omitted from the bias and uncertainty analysis as they were found to be inferior to MONO6 and NCEXP20 fitting methods in the preceding analysis (see Section 3.3). Signal data was simulated to find the bias and uncertainty of the MONO6 and NCEXP20 curve fits over the SNR range from 5 to 40 (steps by 5) and *T*_2_ range from 50 to 200 ms (steps by 10 ms), for 128 combinations of SNR and *T*_2_. Each combination of SNR and *T*_2_ was simulated 100 times (representing 100 voxels), and the MONO and NCEXP fits performed to find *T*_2_ for each simulated voxel. The percent error of each calculated *T*_2_ was then found as $\frac{\left|\text{Calculated}\;T_{2}-\text{Expected}\;T_{2}\right|}{\text{Expected}\;T_{2}}\times 100$, and the percent errors were averaged across simulations for every combination of SNR and *T*_2_. The bias was defined as the average percent error of the 100 voxels for each combo of SNR and *T*_2_. The uncertainty was defined as the SD of the percent error of the 100 voxels. Heatmaps were generated from bias and uncertainty data using MATLAB's *contourf* function. Zero bias or uncertainty was mapped to white, while 50% error was mapped to red, meaning that areas of white indicated an accurate *T*_2_ calculation while areas of red indicated poor accuracy.

Although true *T*_2_ values are not available in vivo so error cannot be determined, resultant *T*_2_ maps were calculated for each fitting method for the entire disc to compare the heterogeneity of the methods. To do this, the entire disc region was defined in MATLAB using the polygon tool, then signal data across echoes for every voxel inside the ROI was curve fit individually with the MONO6 and NCEXP20 methods. From the resulting *T*_2_ values, maps were made such that each voxel was assigned a color based on a jet colormap with a range of *T*_2_ values from 0 to 250 ms (black/dark blue indicated low *T*_2_ and red indicated high *T*_2_). These maps were overlaid on the black and white *T*_2_w MR images to visualize *T*_2_ and its heterogeneity from MONO6 and NCEXP20 fit methods.

**FIGURE 5:**
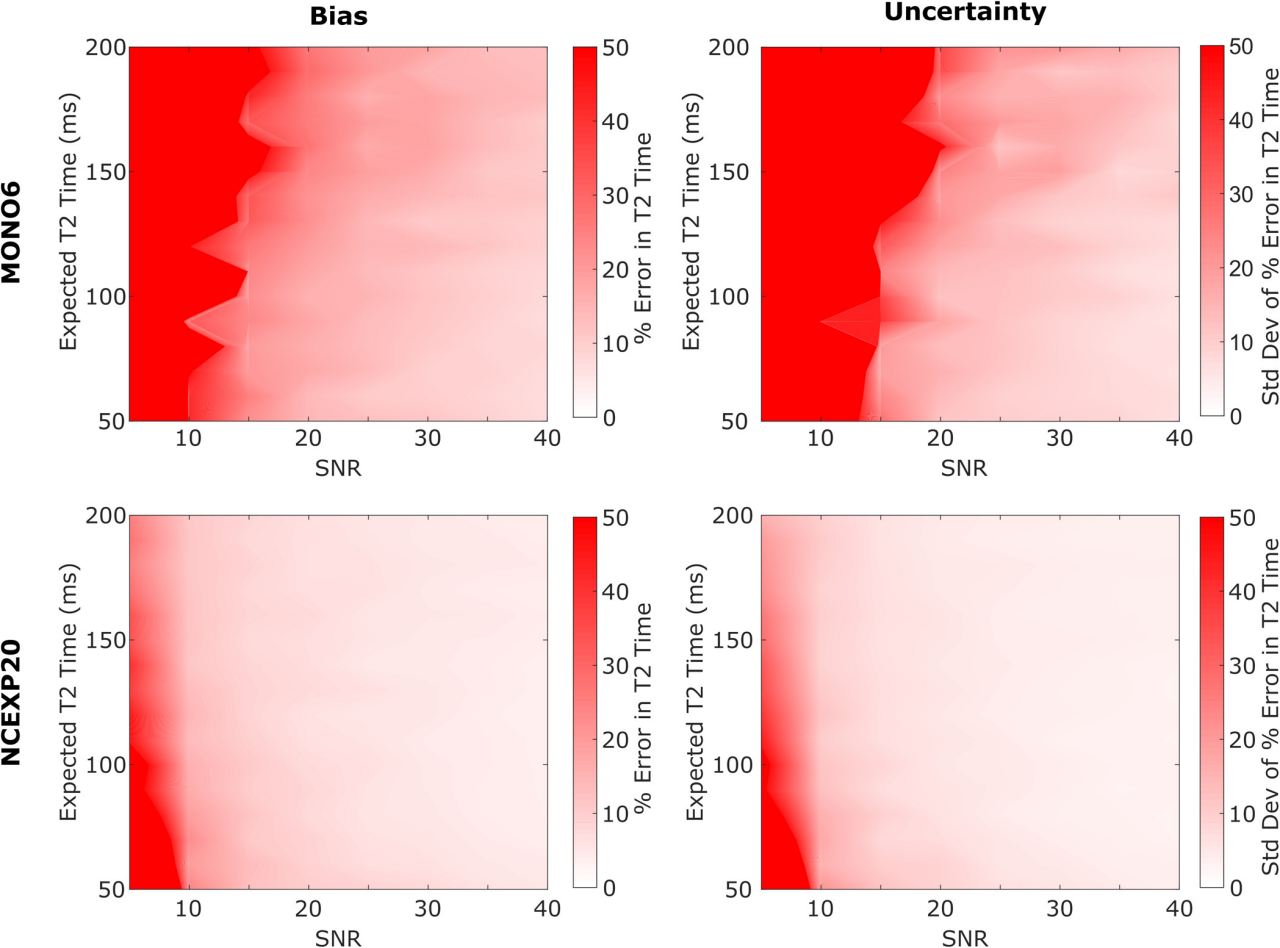
Heatmaps of bias (left) and uncertainty (right) in calculated *T*_2_ for MONO6 (top) and NCEXP20 (bottom) using simulated Rician noise. NCEXP20 has lower bias and uncertainty compared to MONO6, making it a more robust calculation method for finding accurate *T*_2_

MONO6 has higher bias and uncertainty than NCEXP20, particularly at low SNR (Figure 5). The two methods are similar when SNR is greater than 40, but NCEXP20 is still superior. Considering typical disc values, SNR of 10 and *T*_2_ of 130 ms, MONO6 has a bias of 125% while NCEXP20 has a bias of 13.4%. NCEXP20 has a maximum bias of 24.8% at *T*_2_ of 50 ms. Further at SNR of 10, MONO6 bias is >50% error for all but three *T*_2_ values and >100% error for most *T*_2_. Even at SNR of 20, MONO6 exhibits a bias >20% error in half of the *T*_2_, while NCEXP20 has a maximum of 10.1% error. Uncertainty follows the same pattern where MONO6 has much higher maximum uncertainty at low SNR (Figure 5). Overall, NCEXP20 is a more precise and accurate method.

**FIGURE 6:**
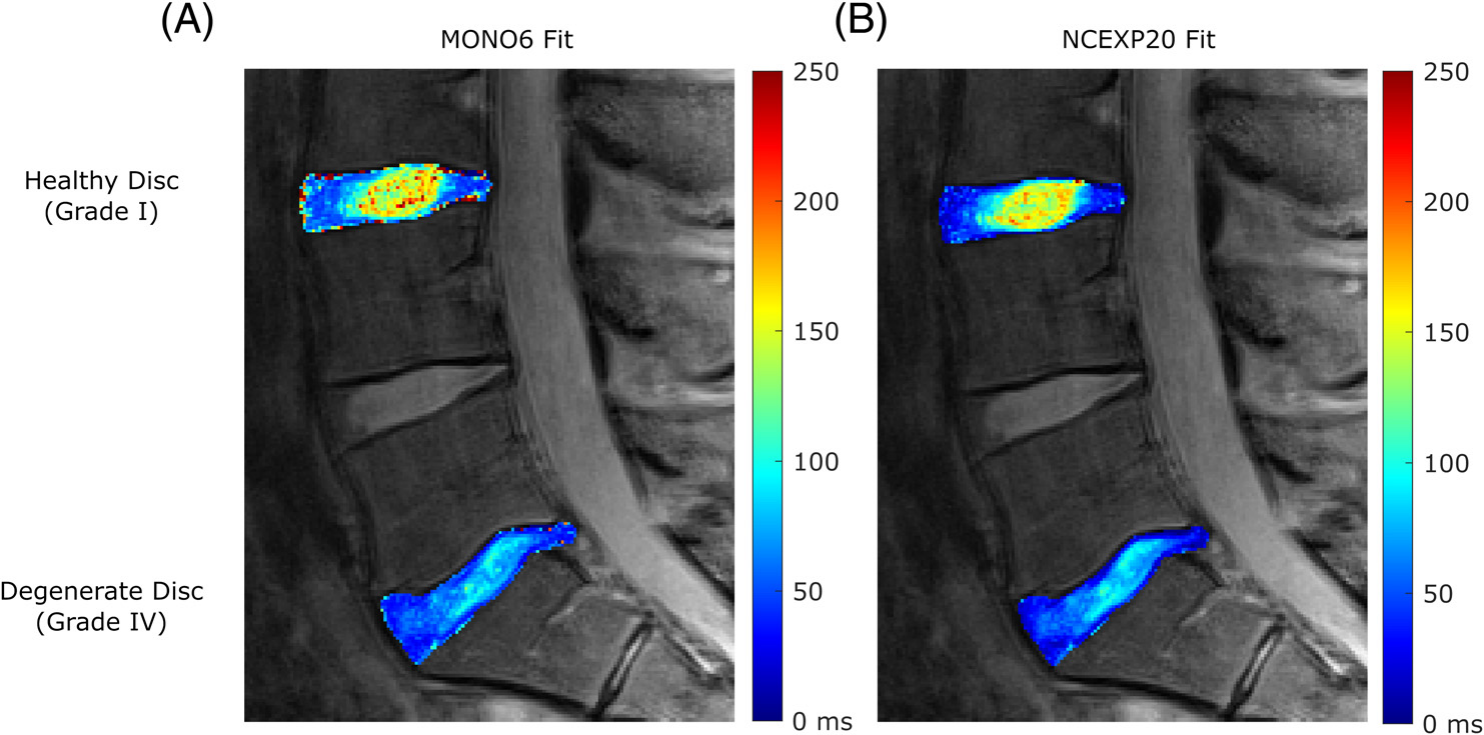
*T*_2_ maps of a healthy L34 disc and degenerate L51 disc with A, MONO6 and B, NCEXP20 fits. NCEXP20 maps are more homogenous with less variation or outlier pixels. MONO6 is more susceptible to error due to noise in signal decay

*T*_2_ maps of full discs allowed visualization of variance of *T*_2_ across the disc (Figure 6). Fits obtained with NCEXP20 display less random variation than those obtained using MONO6. Fitting each voxel with MONO6 shows areas of overestimated *T*_2_, likely outliers, as can be seen by dark red voxels near the edges of the disc and in the NP.These were more frequent in MONO6 fits. MONO6 is much more susceptible to voxels becoming very overestimated or biased, particularly in areas of low signal at the disc edge, but also in the NP. The overall *T*_2_ averaged for all voxels in the disc are impacted by fitting methods. In the healthy disc, where there are larger differences, MONO6 calculates a *T*_2_ of 3547 vs 84 ms for NCEXP20. In the degenerate disc, MONO6 calculates a *T*_2_ of 53 vs 52 ms for NCEXP20. When using the ROI of the NP, the calculated *T*_2_ are more similar. In the healthy disc, MONO6 calculates a *T*_2_ of 155 vs 147 ms with NCEXP20, and in the degenerate disc both curve fits calculate a *T*_2_ of 80 ms. This is likely because the NP has higher SNR compared to the AF and disc edge regions, so there is less bias and fewer outliers. The higher bias in MONO6 at low SNR likely drives the differences between MONO6 and NCEXP20 and results in inaccurate overestimation of *T*_2_.

### 3.5 Comparison of average intensity of ROI vs voxel calculation methods

We also determined if there is a difference between calculated *T*_2_ when first averaging the signal in the ROI and performing a single curve fit to obtain disc *T*_2_ vs performing the curve fit to the signal in each voxel individually and then averaging the *T*_2_ of each within an ROI to obtain disc *T*_2_. ROI results using NCEXP20 curve fitting were taken from section 3.3 and voxel‐wise maps were generated as described in section 3.4. The same circular ROI used in the ROI method in the NP was applied to the disc map from the previous section, and the average *T*_2_ of all voxels inside the ROI was calculated. The resulting distributions of *T*_2_ from all 40 discs were compared with a paired *t* test, and a histogram of the differences between ROI calculation methods was generated as ROI *T*_2_—voxel‐wise *T*_2_.

**FIGURE 7:**
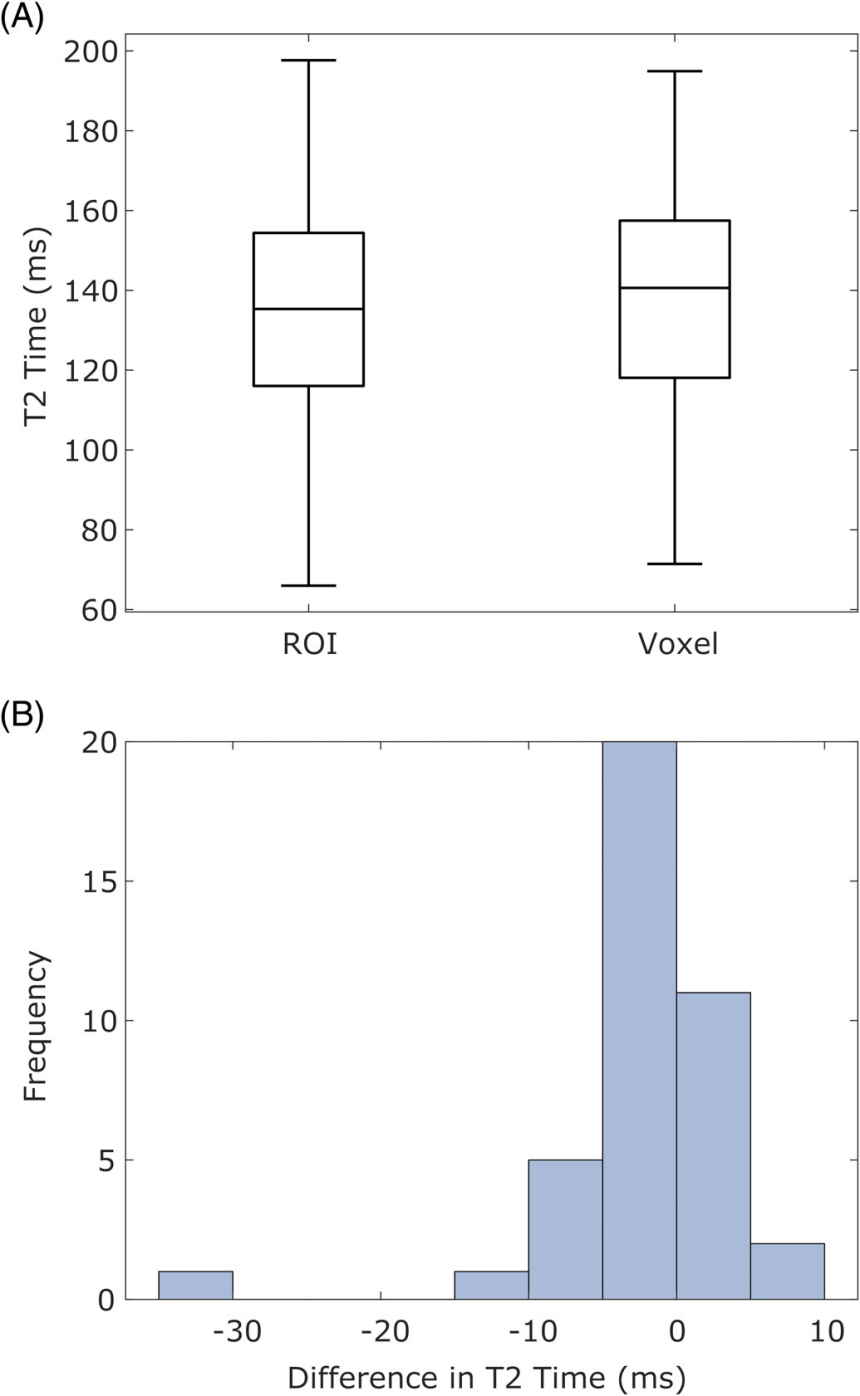
A, Comparison of NCEXP20 for average ROI intensity (left) and Voxel (right) calculation methods for a circular ROI in the NP. Distributions are very similar between methods. B, histogram of the differences between the two ROI methods. Differences are centered around 0 with one outlier that has much higher voxel *T*_2_. Only two discs have *T*_2_ differences greater than 10 ms. Boxes represent median, 25th, and 75th percentiles. Whiskers extend to most extreme data point that is not considered an outlier (+ symbol)

The two methods give very similar results with an average absolute difference of 3.54 ms and average percent difference of 2.77% (*p* > .05, Figure 7A). Of the 40 discs investigated, only two discs exhibited a difference between methods of more than 10 ms (11.19 and 30.23, both Grade I, Figure 7B). The average absolute differences are within the range of standard deviations of reported *T*_2_. Although voxel methods require much more computation time and single voxels may create outliers (Figure 6), both methods should be assumed to be accurate when SNR is reasonable. However, care may need to be taken when the ROI has a small number of voxels or contains very low SNR regions that may create outliers that may skew the data. Of note, the fit for the voxel method required 60 to 300 seconds depending on ROI size, while a single fit for the ROI method takes less than 5 seconds.

## 4 DISCUSSION

This study showed that using the noise corrected exponential (NCEXP) to fit data from 20 echoes out to ~300 ms is the best method for calculating *T*_2_ of discs because it is the least likely to be biased by low SNR. By acquiring data out to longer echo times and accounting for Rician noise, the curve fitting is more robust in calculating *T*_2_ despite the noise in the data. This is particularly important when considering degenerate discs or AF tissue because the SNR of these regions will be lower. Additionally, there is little difference between the calculated *T*_2_ from either averaged intensity fitting or voxel‐wise *T*_2_ calculation, so either method is viable.

NCEXP20 was more accurate at fitting simulated and in vivo data than other fitting methods and had smaller bias and uncertainty compared to MONO6 across all *T*_2_ and SNR combinations. Collecting data out to longer echo times and taking the Rician noise into account during curve fitting resulted in better fitting of signal decay data and more accurate calculation of *T*_2_. In simulated data, at high SNR, either MONO or NCEXP fitting worked reasonably well, as the signal decays to zero and not into the noise floor. But with low SNR, the signal decay is altered by noise and does not decay to zero, so MONO fitting overestimates the *T*_2_. As a result, the difference in fit quality is most noticeable in degenerate discs that have lost water content in the NP and therefore lost signal in *T*_2_w images and have a lowered SNR. Attempting to fit only the first several echoes (representing some studies that do not have sufficient number of echoes in their protocols) with NCEXP was also inaccurate because the data give little information about the eventual noise floor that the NCEXP curve is trying to fit. Thus, using a short *TE* with an acquisition time out to 2× the expected *T*_2_ of the tissue (producing a high number of *TE* and ~85% signal decay) and a NCEXP curve fit is optimal for disc *T*_2_ measurement.

The *T*_2_ values calculated were consistent with previous literature for both healthy and degenerate discs. The healthiest of discs feature NP *T*_2_ in the 150 to 200 ms range, while degenerate grade IV discs were closer to 80 ms. Most literature reports *T*_2_ for healthy discs near 150 ms, while very degenerate discs can be as low as 50 ms.^11^, ^13^, ^14^ Specifically, our data matches closest with literature that has sequence parameters with echoes out to 288 ms and *TR* of 3000 ms.^13^ Based on MR physics and this observation, we recommend sequence parameters of *TR* = 3000 ms (3× the expected *T*_1_ of the tissue) and *TE* out to at least 300 ms (2× the expected *T*_2_ of the tissue). A short *TE* should be utilized to maximize the number of echoes that can be curve fit and to capture quick decaying signal. Some published data report healthy disc *T*_2_ in the 75 to 100 ms range. This is surprisingly low for healthy discs and contrary to our data. These discrepancies could be explained by possible combinations of the low *TR* times leading to signal contamination with *T*_1_ signal, the inclusion of the first echo in the fit, or a low number of echoes in the fit; however, the source of the discrepancy cannot be determined without examining the studies' raw data and curve fits. Old data can be reanalyzed by its owners to determine if systematic *T*_2_ calculation errors occurred, but in the absence of open data we cannot determine if reported *T*_2_ are accurate or proper calculation methods were used. Thus, values reported in the literature should be examined and cautiously used when the methods for calculation are not clear or when methods between papers are not similar.

*T*_2_ can be used as a marker for disc health because of its relation to water content and matrix integrity. As the disc begins to degenerate, the NP loses proteoglycan content and water content. *T*_2_ measurement quantifies the biochemical state because *T*_2_ decreases as water content and water mobility decrease, and thus *T*_2_ serves as a marker for disc degeneration. Further, changes in quantitative *T*_2_ are more robust than simple changes in *T*_2_ weighted signal intensity. It should be noted that it is usually accepted that *T*_2_ does not change very much, if at all, with magnetic field strengths of 3.0T or lower,^26^ though the signal intensity of a disc can vary depending on the scanner, magnetic field strength, coils used, sequence used, temperature of the subject, and many other factors. *T*_2_ is more robust to these factors because it is a signal decay time constant. Last, unlike Pfirrmann grading, *T*_2_ is quantitative, continuous, and objective, all of which are important for a measurement scheme that is intended to be used across studies. Pfirrmann grading has been shown to correlate with *T*_2_ across grades, but *T*_2_ avoids the problem of subjective binning of discs into five grades.^6^, ^11^ Even more importantly, *T*_2_ is easy to calculate, with sequences readily available on clinical scanners, so *T*_2_ measurements can be easily added to existing imaging protocols as a diagnostic tool or for evaluation of treatments.

Our application of NCEXP in disc follows application of this approach in articular cartilage, which generally has a higher water content and higher SNR. The NCEXP fitting with long echo trains is more robust to noise and more accurate in finding *T*_2_ in phantoms and articular cartilage.^19^, ^20^ We applied these methods to the disc in order to improve *T*_2_ calculation in the spine. This approach can be applied to other quantitative MRI methods that are based on fitting monoexponential signal decay (e.g., *T*_1ρ_) and to other fibrous tissues with low SNR (e.g., meniscus or tendon). *T*_1ρ_ also follows a monoexponential signal decay over time and is also susceptible to Rician noise and the presence of a noise floor.^27^ Our pilot studies with agarose phantoms show that NCEXP curve fitting can be applied to *T*_1ρ_ data for better *T*_1ρ_ time calculation at low SNR. Meniscus has very low MR signal because the tightly packed collagen matrix leads to low water mobility and low water content (compared to disc or cartilage), leading to low SNR in the tissue. NCEXP fitting may be an appropriate method to get accurate *T*_2_ or *T*_1ρ_ for this tissue.

Many previous reports calculated *T*_2_ on a voxel basis, but *T*_2_ is often reported for a specific ROI, for example, the NP in the disc, and calculation of an ROI by averaging the signal intensity and only fitting the averaged data once is computationally faster and suppresses effects of noise. On the other hand, voxel‐wise maps are useful for observing inhomogeneities in a region but require a curve fit for every voxel of interest. There appears to be no prior comparison of these two methods in the literature. Depending on the goal of the research, a voxel‐wise method can be used to look at the heterogeneity of a region or for looking at the *T*_2_ of the disc across its width in either the anterior‐posterior or lateral directions. There should be clear lower *T*_2_ regions at the edges of the disc while the NP region will have higher *T*_2_. The differences between the AF and NP may be smaller in less healthy discs. For simply calculating the *T*_2_ of a whole region, the average intensity of the ROI method measures *T*_2_ as robustly as traditional voxel‐wise measures and takes less computation time.

In conclusion, NCEXP curve fitting of long echo trains with short *TE* should be adopted as the primary method for calculating *T*_2_ in the disc. Researchers should use *TR* times that are at least 3000 ms so that nearly full recovery of magnetization is achieved and signal is not contaminated by *T*_1_ weighting. The first echo should be ignored in multi‐echo sequences because of the stimulated echo effect. Either the average ROI intensity method or voxel‐wise method can be used to calculate *T*_2_ depending on the goal of the research, but the average intensity ROI method will be computationally quicker. Moving forward, sequence parameters and calculation methods need to be clearly defined in reports of *T*_2_ in disc and other tissues. If similar methods are adopted by the field, results can be compared more usefully, promoting faster scientific discovery.

## CONFLICT OF INTEREST

We have no conflicts of interest to disclose.

## AUTHOR CONTRIBUTIONS

Kyle D. Meadows, Curtis L. Johnson, and John M. Peloquin contributed to experiments and analysis of the study, Kyle D. Meadows, Curtis L. Johnson, Richard G. Spencer, Edward J. Vresilovic, and Dawn M. Elliott contributed to study design, to results interpretation, and to writing the manuscript. All authors have read and approved submission.

## APPENDIX A

NCEXP function:

NCEXP= @(T2,So,sigma) sqrt(pi*sigma^2/2)*

 exp(-((So*exp(-TE/T2)/(2*sigma)).^2)).*…

 ((1+2*((So*exp(-TE/T2)/(2*sigma)).^2)).*…

 besseli(0,((So*exp(-TE/T2)/(2*sigma)).^2))+…

 2*((So*exp(-TE/T2)/(2*sigma)).^2).*…

 besseli(1,((So*exp(-TE/T2)/(2*sigma)).^2)));

Where TE is a vector of echo times at which signal data was collected.

Cost function:

fun = @(dummy) sum((Data-NCEXP(dummy(1),dummy(2),dummy(3))).^2);

Where “dummy” is a 3 × 1 vector of T2, So, sigma, and “Data” is a vector of signal intensity decay over TE.

Curve fit:

[par_fit, fval] = fmincon(fun,par0,[],[],[],[],lb,ub,[],[]);

Where “par_fit” is a 3 × 1 output vector of fit parameters T2, So, sigma, and “fval” is the value of the objective function for the fit using “par_fit.” “par0” are initial guesses for fit parameters, and “lb” and “ub” are lower and upper bounds of fit parameters, respectively.
